# A fatal case of ANCA-associated vasculitis resulting in multiple organ failure: Case report and literature review

**DOI:** 10.1097/MD.0000000000046937

**Published:** 2026-01-16

**Authors:** Wei Hung Chang, Shih-Chao Chien, Shih-Chun Chien, Ting-Yu Hu

**Affiliations:** a Department of Critical Care Medicine, MacKay Memorial Hospital, Taipei, Taiwan; b Department of Medicine, Mackay Medical College, Taipei, Taiwan; c Department of Emergency Medicine, Mackay Memorial Hospital, Taipei, Taiwan.

**Keywords:** ANCA, diffuse alveolar hemorrhage, hepatorenal syndrome, respiratory failure, vasculitis

## Abstract

**Background:**

Antineutrophil cytoplasmic antibody (ANCA)-associated vasculitis (AAV) may progress rapidly to multi-organ failure.

**Case:**

An 89-year-old woman with diabetes, atrial fibrillation (on rivaroxaban), and chronic kidney disease presented with hemoptysis and respiratory failure. Initially managed as pneumonia, she developed diffuse alveolar hemorrhage, acute kidney injury, and liver dysfunction. p-ANCA positivity confirmed AAV, but immunosuppressive therapy was deferred due to septic shock. Despite aggressive supportive care, she died from multi-organ failure on day 12.

**Conclusion:**

AAV should be considered in elderly patients with hemoptysis and rapid multi-organ deterioration. Early diagnosis and cautious immunosuppressive use are essential for improving outcomes.

## 1. Introduction

Alveolar hemorrhage (AH) often presents clinically as hemoptysis, ranging from a mild, self-limited process to severe, life-threatening acute respiratory failure. Diffuse alveolar hemorrhage (DAH) has a broad differential diagnosis, including primary pulmonary disorders, connective tissue diseases, cardiac structural abnormalities, or coagulation defects.^[1]^ Prompt recognition is crucial because underlying pathologies such as vasculitis can dramatically influence both therapeutic decisions and prognosis.

Antineutrophil cytoplasmic antibody (ANCA)-associated vasculitides (AAVs) comprise 3 major entities – microscopic polyangiitis, granulomatosis with polyangiitis (GPA), and eosinophilic granulomatosis with polyangiitis (EGPA) – each involving small-vessel inflammation and multi-organ manifestations.^[2]^ The pathogenesis of AAV centers on neutrophil extracellular traps. Impaired NET degradation results in the loss of immune tolerance toward myeloperoxidase (MPO) and proteinase 3 (PR3), leading to an autoimmune cascade of neutrophil activation, excessive reactive oxygen species production, and ongoing vascular injury.

Once the pulmonary microvasculature is affected, AAV may manifest as DAH with rapid progression and high mortality. Clinical data from Geetha and Jefferson emphasized the systemic nature of AAV and the importance of differentiating its subtypes based on antigen specificity, which strongly affects clinical outcomes.^[3]^ Watts and Robson subsequently clarified the classification and epidemiology of vasculitides, underscoring that early recognition of small-vessel vasculitis is essential to prevent irreversible organ damage.^[4]^

Here, we report an elderly patient presenting with acute hemoptysis and respiratory failure who was later diagnosed with p-ANCA–associated vasculitis. In clinical practice, identifying antigen specificity (MPO vs PR3) and antibody titers refines both diagnosis and prognosis. Although such data were unavailable in our case, the coexistence of p-ANCA positivity, DAH, and rapidly progressive renal impairment strongly suggested microscopic polyangiitis.

Recent international guidelines, including the 2021 American College of Rheumatology/Vasculitis Foundation (ACR/VF) recommendations by Chung et al^[5]^ and the 2022 EULAR update by Hellmich et al,^[6]^ emphasize rapid diagnosis, early induction therapy using rituximab or cyclophosphamide, and structured glucocorticoid tapering. These evolving recommendations highlight the critical importance of prompt recognition and individualized immunosuppressive strategies in AAV.

## 2. Case presentation

An 89-year-old woman with a past medical history of:

1. Type 2 diabetes mellitus
2. Atrial fibrillation under rivaroxaban 10 mg/d
3. Hypertension
4. Chronic kidney disease, stage 3
5. Intestinal lymphangiectasia
6. Hyperthyroidism
7. Congestive heart failure with pulmonary hypertension

presented to the emergency department with acute hemoptysis (2 moderate episodes within 1 h) and respiratory distress. Her vitals at presentation were: temperature 36.7°C, heart rate 60 beats/min, respiratory rate 22 breaths/min, blood pressure 178/81 mm Hg, and SpO_2_ 89% on room air. She was fully conscious (GCS E4V5M6). Supplemental oxygen via nasal cannula at 4 L/min was provided, and her airway was protected.

**Figure 1.**
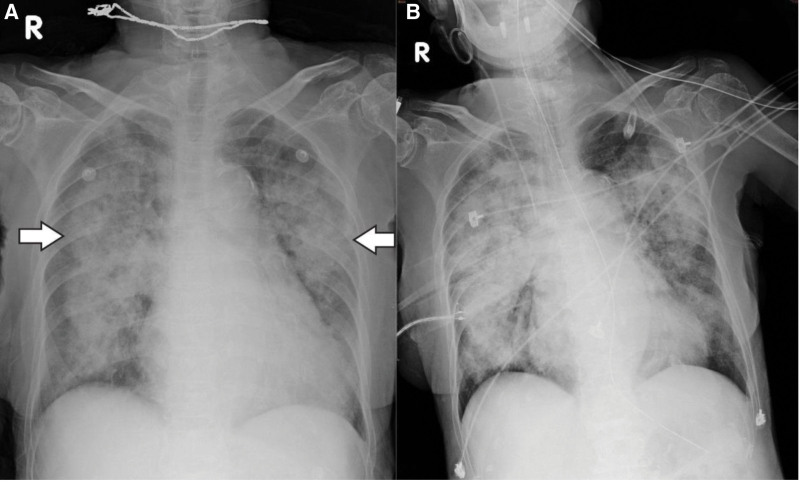
Chest plain film in the emergency department, revealing large bilateral consolidations suspicious for pulmonary edema or pneumonia.

**Figure 2.**
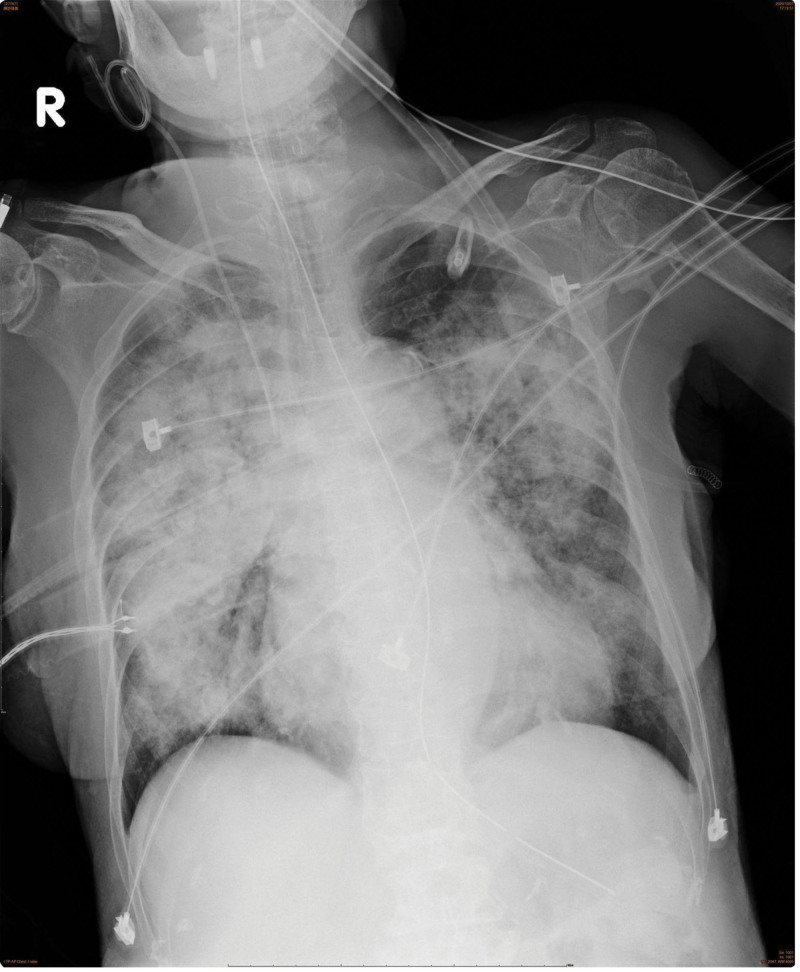
Contrast-enhanced chest CT showing an enlarged main pulmonary artery and extensive bilateral lung consolidations, initially interpreted as pulmonary edema or pneumonia. CT = computed tomography.

Physical examination revealed no gum bleeding, ecchymosis, petechiae, or abdominal tenderness. Initial laboratory data showed leukocytosis (WBC 12,900/μL, neutrophils 87.9%), anemia (Hb 7.2 g/dL), mild coagulopathy (PT 15.4 s, INR 1.54), mildly elevated liver enzymes (AST 57 IU/L, ALT 51 IU/L), creatinine 1.5 mg/dL, and elevated inflammatory markers (CRP 3.901 mg/dL, procalcitonin 0.32 ng/mL). On 4 L/min O_2_, her arterial blood gas showed pH 7.510, PaCO₂ 37 mm Hg, PaO_2_ 100 mm Hg, HCO_3_ 30 mmol/L. A chest radiograph (Fig. 1) and subsequent CT (Fig. 2) revealed bilateral lung consolidations, raising suspicion of pneumonia or pulmonary edema. Rivaroxaban was held, tranexamic acid 250 mg IV was administered, and 2 units of leukocyte-poor packed red blood cells were transfused.

## 3. Results/clinical course

The patient’s ICU stay lasted 12 days. A day-by-day summary of clinical progression is presented below:

- Day 1 (10/7): Intubated for severe hypoxemia (PaO₂/FiO₂ ≈ 70 on 100% O₂), transferred to ICU. Bronchoscopy showed diffuse bloody secretions, consistent with DAH. Initial autoimmune workup sent.
- Day 2 to 3: Persistent hemoptysis and high FiO₂ requirement (80–100%). Inhaled epinephrine and tranexamic acid were continued. Procalcitonin rose from 0.32 to 1.30 ng/mL.
- Day 4 (10/10): Jaundice progressed; laboratory testing showed worsening direct hyperbilirubinemia (DB 5.3 mg/dL) and elevated liver enzymes (AST 303 IU/L, ALT 168 IU/L). Ultrasound revealed congestive hepatopathy.
- Day 5 (10/11): Oxygenation remained poor (FiO₂ 90%), vasopressor support initiated for borderline hypotension.
- Day 6 (10/12): Septic shock developed, requiring norepinephrine infusion. Procalcitonin increased to 1.86 ng/mL. The suspected infection source was pulmonary, although no definitive pathogen was isolated in the available records. Serial procalcitonin rose (0.32 → 1.30 → 1.86 → 4.59 ng/mL), supporting active infection despite negative culture documentation.
- Day 7 (10/13): Progressive renal dysfunction (Cr 3.7 mg/dL, BUN 73 mg/dL); continuous venovenous hemofiltration initiated. Autoimmune workup later confirmed p-ANCA positivity, c-ANCA negative.

Autoimmune workup revealed p-ANCA positivity (qualitative immunoassay), c-ANCA negative. Quantitative titers were not available at the time of diagnosis; however, the positive result, combined with the clinical context of DAH, progressive renal dysfunction, and exclusion of alternative etiologies, supported, but did not definitively confirm, the diagnosis of ANCA-associated vasculitis.

- Day 8 to 10 (10/14–10/16): Despite broad-spectrum antibiotics (meropenem + tigecycline), FiO₂ demand remained 80% to 90%. Chest radiography showed only partial improvement. Procalcitonin continued to rise, peaking at 4.59 ng/mL.
- Day 11 (10/17): Persistent high-dose vasopressor requirement (norepinephrine up to 6 cc/hr). Antibiotics discontinued after prolonged courses. Repeat ANCA sent.
- Day 12 (10/18): Hemodynamic collapse with bradycardia and refractory hypotension, leading to death despite maximal support.

**Figure 3.**
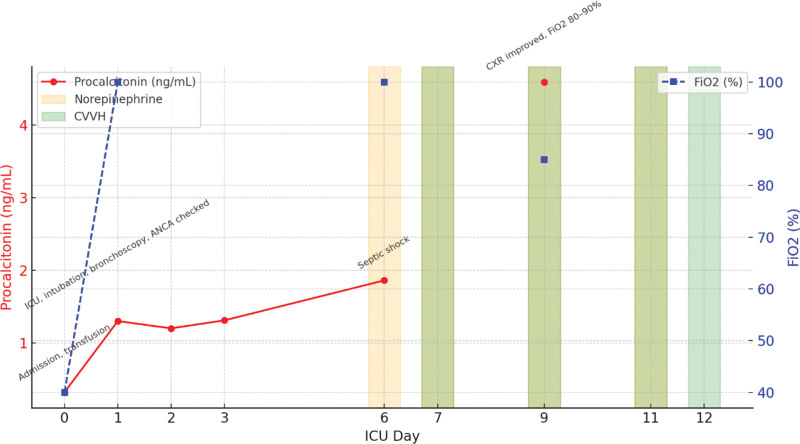
Simplified ICU timeline (day 0–12) showing procalcitonin trajectory, FiO₂ demand, norepinephrine use, initiation of CVVH, antibiotic discontinuation, and major events (bronchoscopy, septic shock, imaging improvement, DNR, and death). Exact daily PaO₂/FiO₂ ratios and continuous vasopressor dosages were not available in the records. CVVH = continuous venovenous hemofiltration, DNR = do not resuscitate.

A detailed ICU timeline of ventilatory parameters, vasopressor use, renal replacement therapy, and inflammatory markers is summarized in Table 1 and illustrated in Figure 3.

**Table 1:** ICU daily timeline showing 12-day progression of ventilatory status, vasopressor use, renal replacement therapy, and inflammatory markers.

Day	Ventilation/oxygenation	Hemodynamics	Renal support	Infection/inflammation	Key events
1 (10/7)	Intubated, PaO₂/FiO₂ ~70 (100% O₂); bronchoscopy: DAH	Stable	–	PCT 0.32 ng/mL	ICU admission
2–3	FiO₂ 80–100%; hemoptysis persisted	Stable	–	PCT 1.30 → 1.20 ng/mL	Inhaled epinephrine and tranexamic acid
4 (10/10)	FiO₂ ~90%	Stable	–	DB 5.3 mg/dL; AST 303; ALT 168	Jaundice, congestive hepatopathy
5 (10/11)	FiO₂ ~90%	Vasopressor support started	–	–	Borderline hypotension
6 (10/12)	FiO₂ 100%; severe hypoxemia	Septic shock → norepinephrine	–	PCT 1.86 ng/mL	–
7 (10/13)	FiO₂ >90%	On norepinephrine	CVVH initiated	Cr 3.7 mg/dL; BUN 73 mg/dL	p-ANCA (+), c-ANCA (–)
8–10	FiO₂ 80–90%	High-dose vasopressors	CVVH ongoing	PCT peak 4.59 ng/mL	CXR partial improvement
11 (10/17)	FiO₂ 85%	Norepinephrine 6 cc/hr	CVVH ongoing	–	Antibiotics discontinued; repeat ANCA sent
12 (10/18)	FiO₂ 90%	BP drop, bradycardia → asystole	–	–	Death

ALT = alanine aminotransferase, ANCA = anti-neutrophil cytoplasmic antibody, AST = aspartate aminotransferase, BUN = blood urea nitrogen, Cr = creatinine, CVVH = continuous venovenous hemofiltration, CXR = chest X-ray, DAH = diffuse alveolar hemorrhage, DB = direct bilirubin, PCT = procalcitonin.

A comprehensive summary of hematological, biochemical, and immunological parameters is provided in Table 2, highlighting the temporal progression of inflammation, renal and hepatic dysfunction, and autoimmune markers during the ICU stay.

**Table 2:** Laboratory and immunological parameters during ICU course.

Day	Hematology/inflammation	Renal function	Liver function	Immunological tests
1 (10/7)	WBC 12,900/µL; Hb 7.2 g/dL; CRP 3.9 mg/dL; PCT 0.32 ng/mL	Cr 1.5 mg/dL	AST 57 IU/L; ALT 51 IU/L	ANA < 1:80; RF < 10 IU/mL
2–3	Hb stable post transfusion; PCT 1.30 → 1.20 ng/mL	–	–	Lupus anticoagulant (+); C3 63 mg/dL; C4 15 mg/dL
4 (10/10)	PCT 1.31 ng/mL	–	DB 5.3 mg/dL; TB 7.7 mg/dL; AST 303; ALT 168	–
6 (10/12)	PCT 1.86 ng/mL	Cr 3.7 mg/dL; BUN 73 mg/dL	–	–
7 (10/13)	Hb ↓; PCT ~2–3 ng/mL	Cr 3.7 mg/dL; BUN 73 mg/dL	–	p-ANCA (+), c-ANCA (–)
8–10	PCT peak 4.59 ng/mL	CVVH ongoing	–	–
11 (10/17)	–	CVVH ongoing	–	Repeat ANCA sent
12 (10/18)	–	–	–	–

ANA = antinuclear antibody, ANCA = anti-neutrophil cytoplasmic antibody, BUN = blood urea nitrogen, Cr = creatinine, CRP = C-reactive protein, CVVH = continuous venovenous hemofiltration, DB = direct bilirubin, PCT = procalcitonin, RF = rheumatoid factor, TB = total bilirubin.

Key laboratory and clinical parameters across the ICU stay are summarized in Table 2. A more detailed listing of hematological, biochemical, and immunological results is provided in Table S1, Supplemental Digital Content, https://links.lww.com/MD/R91.

## 4. Discussion

In an elderly patient with extensive comorbidities, hemoptysis can arise from numerous etiologies, including congestive heart failure, pneumonia, structural lung disease, or vasculitis. Chronic rivaroxaban use could mask or worsen a hemorrhagic process. Several reports have described DAH linked to direct oral anticoagulants, particularly rivaroxaban, in elderly patients with multiple comorbidities. Clinical features often mimic infection or vasculitis, presenting with bilateral infiltrates and acute hemoglobin decline. In our case, rivaroxaban could have contributed to the initial hemorrhage, further confounding early differentiation from ANCA-associated DAH. This overlap underscores the importance of integrating medication history with immunological testing when evaluating hemoptysis in high-risk patients. This case highlights the diagnostic challenge of recognizing ANCA vasculitis when multiple factors – coagulopathy, infection, pulmonary hypertension, renal impairment – overlap.

In this patient, p-ANCA was detected qualitatively, while quantitative titers were not reported. Although the absence of quantitative data limited disease activity assessment, the constellation of DAH, rapidly progressive renal impairment, and hepatic congestion favored a systemic vasculitic process. Severe pulmonary hypertension with congestive hepatopathy can mimic sepsis- or cardiac-related liver dysfunction, adding diagnostic complexity. Nevertheless, the temporal correlation of ANCA positivity with clinical deterioration reinforced the working diagnosis of AAV.

Infectious considerations. The decision to defer immunosuppressive therapy was driven by ongoing septic shock physiology. Although no culture-proven pathogen was available in the records, the clinical picture was consistent with pulmonary sepsis, and procalcitonin rose steadily from 0.32 ng/mL at admission to 4.59 ng/mL before death. This biomarker trajectory, together with hemodynamic collapse and vasopressor dependence, provided a strong rationale for prioritizing infection control and supportive measures over immediate immunosuppression.

Therapeutic trade-offs. Managing fulminant AAV in the setting of uncontrolled sepsis presents a critical dilemma. Guidelines for organ- or life-threatening AAV recommend high-dose glucocorticoids combined with cyclophosphamide or rituximab; however, such therapy was contraindicated here due to ongoing septic shock. Plasma exchange (PLEX) represents a theoretical alternative, as it can remove circulating ANCA and inflammatory mediators without broad immunosuppression. Nevertheless, evidence from the PEXIVAS trial did not demonstrate a significant mortality or end-stage renal disease benefit of routine PLEX in severe AAV, suggesting selective rather than universal use. In our patient, persistent hemodynamic instability, continuous renal replacement therapy already in place, and a family decision to decline further invasive interventions precluded escalation to PLEX. This highlights the importance of individualized decisions, balancing immunologic control against infection risk and procedural burden. Our management considerations align with the recent ACR/VF (2021) and EULAR (2022) guidelines, which caution that immunosuppressive strategies must be adapted when infection risk is high, and that plasma exchange should be reserved for selected catastrophic cases rather than used routinely.

Pulmonary arterial hypertension (PAH) secondary to AAV is known but often underrecognized; it can mimic idiopathic PAH, as reported by Pilania et al,^[7]^ Baqir et al,^[8]^ and Yoshifuji et al.^[9]^ Similar to other connective tissue diseases such as systemic lupus erythematosus, immune-mediated vasculopathy can cause PAH, and immunosuppression may stabilize or reverse these changes, as summarized by Aithala et al.^[10]^

Pathophysiologically, AAV-associated pulmonary hypertension is believed to result from small-vessel vasculitis, capillaritis, and chronic parenchymal remodeling after repeated episodes of DAH, in contrast to the plexogenic arteriopathy typical of idiopathic PAH. Clinically, AAV-related PH often coexists with interstitial lung disease or sequelae of repeated DAH, making it more heterogeneous than idiopathic forms. These distinctions explain why vasculitis-related PH may partially respond to immunosuppressive therapy, unlike idiopathic PAH where vasodilator therapy remains the mainstay.

Imaging features such as ground-glass opacities and cavitating nodules can indicate active vasculitis, as described by Schmidt.^[11]^ However, the overlap with infection or aspiration is common and confounding, as demonstrated by Yoo et al^[12]^ and Buhaescu et al.^[13]^

Pulmonary–renal syndrome (PRS), characterized by DAH and rapidly progressive glomerulonephritis, remains one of the most severe manifestations of AAV. Battisha et al reported a hydralazine-induced case presenting with PRS,^[14]^ while Agarwal et al^[15]^ and Marina et al^[16]^ also documented similar ANCA-associated vasculitic presentations leading to severe renal and pulmonary injury. Treatment options include corticosteroids, cyclophosphamide, rituximab, and plasmapheresis, with outcomes ranging from complete recovery to chronic dialysis dependence.

However, superimposed sepsis made immunosuppression prohibitive in this patient, illustrating a frequent dilemma in managing AAV exacerbations complicated by infection.

Although high-efficiency hemodiafiltration and plasmapheresis are recognized therapeutic options for ANCA-associated vasculitis with DAH or PRS, their application was limited in this patient. Continuous venovenous hemofiltration was initiated for progressive renal dysfunction, but the patient’s concurrent septic shock and multiorgan failure markedly increased the risks associated with further extracorporeal therapies. Moreover, the family declined additional invasive interventions after signing a do-not-resuscitate order. In this context, escalation to plasmapheresis or high-flux HDF was not pursued. This decision is consistent with findings from the PEXIVAS trial, which showed no significant mortality or end-stage renal disease benefit of plasma exchange in most AAV patients, though individual cases may still warrant consideration.

## 5. Conclusion

This case demonstrates a challenging clinical scenario in which an elderly patient presented with hemoptysis and rapidly evolved to life-threatening respiratory, hepatic, and renal failure. The late recognition of ANCA-associated vasculitis underscores the need for early diagnostic consideration of vasculitis in acute hemorrhagic or multi-organ processes. Timely immunosuppressive therapy, if balanced against infection risk, may improve outcomes. When culture results are unavailable, integrating infection biomarkers such as procalcitonin can help guide the balance between infection control and timely immunosuppression. The patient’s hepatic and renal dysfunction were considered part of sepsis-associated multiorgan dysfunction rather than classical hepatorenal syndrome. However, uncontrolled sepsis can preclude appropriate vasculitis treatment, highlighting the importance of vigilance and comprehensive diagnostic workups in high-risk populations.

Clear reporting of ANCA antigen specificity (MPO/PR3) and titers, when available, is essential to interpret treatment decisions and outcomes in similar scenarios.

## Acknowledgments

We would like to thank Dr Yu-Yi Chien for his expert advice and encouragement throughout this study.

## Author contributions

**Conceptualization:** Shih-Chao Chien, Shih-Chun Chien.

**Supervision:** Ting-Yu Hu.

**Writing – review & editing:** Wei Hung Chang.

## Supplementary Material


