# Supplementary material for: A fatal case of ANCA-associated vasculitis resulting in multiple organ failure: Case report and literature review
Source: Medicine (Baltimore). 2026 Jan 16;105(3):e46937. doi: 10.1097/MD.0000000000046937 (PMC12826183; doi:10.1097/MD.0000000000046937)
Supplement: Supplementary file 1 [file medi-105-e46937-s001.docx]

**Supplementary Table S1**（Online supplement）. Key laboratory and clinical trends during ICU stay

| **Day** | **Hb (g/dL)** | **Cr (mg/dL)** | **BUN (mg/dL)** | **AST/ALT (IU/L)** | **DB (mg/dL)** | **CRP (mg/dL)** | **PCT (ng/mL)** |
| --- | --- | --- | --- | --- | --- | --- | --- |
| ED (10/6) | 7.2 | 1.5 | NA | 57 / 51 | NA | 3.9 | **0.32** |
| Day 1 (10/7) | NA | NA | NA | NA | NA | NA | **1.30** |
| Day 2–3 | NA | NA | NA | NA | NA | NA | 1.20–1.31 |
| Day 4 (10/10) | NA | NA | NA | **303 / 168** | **5.3** | NA | NA |
| Day 6 (10/12) | ↑ | ↑ | ↑ | NA | NA | NA | **1.86** |
| Day 7 (10/13) | NA | 3.7 | 73 | NA | NA | NA | NA |
| Day 8–10 | NA | NA | NA | NA | NA | NA | **4.59 (peak)** |
| Day 11 (10/17) | NA | NA | NA | NA | NA | NA | NA |
| Day 12 (10/18) | NA | NA | NA | NA | NA | NA | NA |

NA = not available in records. ↑ = progressive rise.
